# Population Connectivity Measures of Fishery-Targeted Coral Reef Species to Inform Marine Reserve Network Design in Fiji

**DOI:** 10.1038/srep19318

**Published:** 2016-01-25

**Authors:** Erin K. Eastwood, Elora H. López, Joshua A. Drew

**Affiliations:** 1 Department of Ecology, Evolution, and Environmental Biology, Columbia University, NY; 2 Department of Vertebrate Zoology, American Museum of Natural History, NY

## Abstract

Coral reef fish serve as food sources to coastal communities worldwide, yet are vulnerable to mounting anthropogenic pressures like overfishing and climate change. Marine reserve networks have become important tools for mitigating these pressures, and one of the most critical factors in determining their spatial design is the degree of connectivity among different populations of species prioritized for protection. To help inform the spatial design of an expanded reserve network in Fiji, we used rapidly evolving mitochondrial genes to investigate connectivity patterns of three coral reef species targeted by fisheries in Fiji: *Epinephelus merra* (Serranidae)*, Halichoeres trimaculatus* (Labridae), and *Holothuria atra* (Holothuriidae). The two fish species, *E. merra* and *Ha. trimaculatus*, exhibited low genetic structuring and high amounts of gene flow, whereas the sea cucumber *Ho. atra* displayed high genetic partitioning and predominantly westward gene flow. The idiosyncratic patterns observed among these species indicate that patterns of connectivity in Fiji are likely determined by a combination of oceanographic and ecological characteristics. Our data indicate that in the cases of species with high connectivity, other factors such as representation or political availability may dictate where reserves are placed. In low connectivity species, ensuring upstream and downstream connections is critical.

Coral reefs are some of the planet’s most complex and diverse ecosystems, providing nearly US$ 30 billion in goods and services to global economies annually, through tourism, fisheries, and coastal protection^1^. However, the vivid splendor of these ecosystems is subject to numerous anthropogenic stressors including overfishing, pollution, sedimentation, and climate change, and nearly every coral reef worldwide is currently affected by human activities^2^,^3^,^4^.

One tool commonly used to mitigate global reef degradation is the creation of marine protected areas (MPAs) – spatially explicit areas of ocean where human activities are regulated or prohibited. One subset of MPAs are no-take marine reserves, wherein direct harvesting of marine resources is prohibited both spatially and temporally. When effectively implemented, no-take marine reserves are important tools for conservation and fisheries management. They provide a refuge for exploited species, allow for fish and invertebrate stocks to recover, reproduce, and reseed adjacent unprotected areas through larval export and adult movement, while they protecting existing habitat from further degradation^5^,^6^,^7^,^8^.

Networks of reserves confer additional benefits, protecting a greater diversity of habitats and promoting the stability of meta-populations^9^,^10^. The optimal orientation for reserve networks requires information concerning the degree of demographic or larval exchange occurring between populations^11^,^12^. Reserve networks that explicitly incorporate connectivity in their design are more resilient to threats like overfishing, disease, and climate change because neighboring reserves can help reseed reefs in the event of disturbances, thereby boosting the stability of the system as a whole^11^,^13^,^14^.

Species’ behaviour, locomotion, life history, and habitat all play into how organisms interact with their physical environment, and thus influence connectivity. The larvae of many reef fishes possess an array of sensory modalities and the physiological capacity for directional swimming that allows for non-random settlement^15^,^16^,^17^,^18^, and some species with prolonged pelagic larval stages display natal reef preference and genetic structuring across populations^18^,^19^. Despite the capabilities of some larvae, oceanic currents can limit genetic connectivity between populations if individuals are unable to traverse those currents, as this reduces gene flow^20^,^21^,^22^,^23^. Conversely, ocean currents can promote connectivity if they transport larvae from upstream to downstream sites, thus generating asymmetrical gene flow^24^.

In 2005, the Fiji government set the goal of protecting 30% of its marine waters by 2020^25^. As of 2010, local management had effectively protected a considerable proportion of Fiji’s priority ecosystems for management^26^. However, the amount of area that can be protected by ad-hoc community-based management is nearing saturation, and these efforts need to be scaled up significantly if the national goal is to be reached^26^. Because of this, a systematic conservation planning approach that includes consideration of the connectivity and complementarity of potential reserve sites across the entirety of Fiji has been recommended^27^. Additional scientific input, particularly information concerning population connectivity, will be necessary if an expanded network of MPAs is to effectively utilize limited conservation resources in the country.

Previous work on connectivity among reefs in Fiji revealed asymmetrical gene flow along an east-west gradient for three of five coral reef fish species studied, potentially indicating that the Bligh Waters – a fast-moving current that bisects the main islands – could be facilitating larval transport for multiple taxa within Fiji^28^. However, this study was based on a suite of species that were not fisheries targets. A more relevant framework for conservation planning might instead be crafted based on the connectivity dynamics of economically and nutritionally significant species. Small-scale inshore fisheries provide food for about 50% of rural households in Fiji and contribute over $48 million to the national economy every year^29^,^30^, so the sustainable management of species contributing to these fisheries is paramount for ensuring food security. Here, we expand upon previous work and investigate the role of the Bligh Waters in shaping the connectivity patterns of three coral reef species commonly targeted by inshore fisheries across the Fijian archipelago (Fig. 1) – the honeycomb grouper *Epinephelus merra* (Serranidae), the three-spot wrasse *Halichoeres trimaculatus* (Labridae), and the black sea cucumber *Holothuria atra* (Holothuriidae).

## Results

### Genetic Diversity

We sequenced 475 bp of the mtDNA control region from 82 individuals of *E. merra*, 376 bp of the same locus from 91 individuals of *Ha. trimaculatus*, and 376 bp of the COI gene from 40 individuals of *Ho. atra* (Table 1). Both fish species displayed high haplotype diversity and had highly polymorphic control regions (87 and 71 polymorphic loci within *E. merra* and *Ha. trimaculatus* sequences, respectively), while *Ho. atra* COI sequences were considerably less genetically diverse (Table 2) and had only 15 polymorphic loci.

### Population Structure and Power Analyses

*E. merra* was characterized by very low and non-significant measures of genetic differentiation at the island level (pairwise **Φ**_ST_ values in Table 3A), after an AMOVA grouping the two Vanuabalavu sites together (Naracivo and Donicali) resulted in non-significant F-statistics for among-group (F_CT_), within-group (F_SC_), and within-population (F_ST_) genetic differentiation (Table 4A). Sampling locations were also assembled into 4 groups that reflect the political units of Fiji (e.g. grouping Nagigi and Naselesele together, as they both belong to the Northern Division), and this second AMOVA also revealed non-significant F-statistics for each hierarchical level (Table 4B).

*Ha. trimaculatus* displayed slightly different patterns of genetic partitioning. This species’ pairwise **Φ**_ST_ values were similarly low but exhibited significant north-south genetic partitioning between the Vanua Levu village of Nagigi and the Viti Levu village of Nabukavese (**Φ**_ST_ = 0.07 p = 0.032, Table 3B). An AMOVA clustering *Ha. trimaculatus* populations into 4 groups reflecting political units within Fiji also revealed non-significant F-statistics for each hierarchical level (Table 4C).

Analysis of *Ho. atra* populations returned a wide range of pairwise **Φ**_ST_ values (Table 3C). Very low values were observed between Taveuni and both the eastern island of Vanuabalavu and the western island and Naviti, and relatively high values between Vanua Levu and all other island sites. The highest pairwise **Φ**_ST_ value occurred between Vanua Levu and Vanuabalavu. Because genetic partitioning was detected between the same-Division islands of Vanua Levu and Taveuni at this initial step, an AMOVA further grouping locations together was not performed. Further, the majority of *Ho. atra* samples (n = 35) fit into one of three haplotypes (Fig. 2). The most highly represented haplotype (n = 29) was found in individuals from all four sampling locations. The western island of Naviti had the highest number of individuals with private alleles, and the eastern island of Vanuabalavu had the lowest measures of intra-population genetic diversity.

Results of the statistical power analyses revealed that in both fish species, the control region markers had sufficient power to detect genetic differentiation above a **Φ**_ST_ value of 0.01, which corresponds with low levels of genetic differentiation and high amounts of connectivity (Fig. 3)^31^.

### Migration Estimates

MIGRATE analyses resolved high levels of migration but with complex patterns of directionality among populations. For *E. merra*, the individuals from Nabukavesi, located on the southern large island of Viti Levu appear to be a major source population, providing high numbers of migrants per generation to other localities – in some cases, up to 10 times the amount migrants received (Table 5). These patterns are the opposite of those observed in *Ha. trimaculatus* – with the site on northern large island of Vanua Levu serving as a source and Viti Levu as a sink (Table 5). Additionally, in both species the western island of Naviti provided more migrants per generation to the far eastern island of Vanuabalavu than vice versa (3.5 times more in *E. merra*, 2.5 times more in *Ha. trimaculatus*).

MIGRATE analyses of *Ho. atra* sequences showed that three of the five highest migration values were from the eastern island of Vanuabalavu out to more western sites. The highest estimated median number of migrants per generation was from Vanuabalavu to Taveuni (672.3), while the lowest estimated number of migrants per generation was from Malevu to Vanuabalavu (388.3-Table 5). There were more westward moving migrants than eastward moving migrants for four of the six pairs of sites. (Table 3).

Tajima’s D values were negative for both *E. merra* and *Ha. trimaculatus* (−1.15228 and −1.20336, respectively), and positive for *Ho. atra* (0.71194). All values were non-significant (p = 0.109, p = 0.094, p = 0.712, respectively).

Finally, the Mantel test revealed negative or flat levels of correlation between pairwise measures genetic differentiation and geographic distance in all three species (*E. merra* R^2^ = 0.001, *Ha. trimaculatus* R^2^ = 0.005, *Ho. atra* R^2^ = 0.010), indicating that none of these species’ patterns of genetic differentiation can be explained by geographic distance between populations.

## Discussion

Different patterns of connectivity were observed for each of the three species in this study, highlighting the complexity of larval transport and population demography in the marine environment. High amounts of gene flow and very low amounts of genetic structure were observed for both fish species, indicating that on the relatively small spatial scale of the Fijian archipelago, populations of *E. merra* and *Ha. trimaculatus* are interconnected. High gene flow is a common phenomenon in marine species, as the marine environment has relatively few physical barriers and long pelagic larval durations often promote long-distance dispersal^32^,^33^,^34^.

The high level of connectivity we observed among populations of *E. merra* is not surprising, and mirrors connectivity levels found in the Philippines^35^ and the Western Indian Ocean^36^. *Ha. trimaculatus* generally exhibited high levels of connectivity among populations as well, but this species did show a subtle yet significant amount of genetic partitioning between the northern island of Vanua Levu and southern island of Viti Levu. These findings support the hypothesis that the Bligh Waters may be a major oceanographic feature affecting connectivity in Fiji – a |pattern observed in a previous comparative phylogeographic study^28^.

Fast-flowing currents like the Bligh Waters can act as barriers to dispersal by advecting larvae out of the system before they are able to settle on reefs opposite of the current, thus preventing genetic exchange between populations^20^,^21^,^22^,^23^. *Ha. trimaculatus*’ low amount of genetic partitioning may indicate that the Bligh Waters has been acting as an oceanographic barrier for a relatively short evolutionary time scale, or that historical factors could also have contributed to this species’ subtle phylogeographic structure – for instance, sea level shifts influencing habitat availability^37^.

In contrast, the sea cucumber *Ho. atra* exhibited complex patterns of connectivity among the four populations sampled. There was little to no genetic differentiation between Taveuni and the islands of the Lau group, but high amounts of partitioning between Taveuni and Vanua Levu despite these two locations’ geographic proximity. The populations in the Lau group were also genetically divergent from those in Vanua Levu. This may be due to oceanographic characteristics of the Somosomo Strait, the narrow channel that passes between the islands of Taveuni and Vanua Levu. More oceanographic investigation is necessary to understand if larvae spawned at Taveuni are brought out to sea by a current that bypasses the reefs on the southern edge of Vanua Levu. Skillings et al. 2011 found *Ho. atra* to be genetically differentiated across distances comparable to that between Vanua Levu and Taveuni (74.8 km), suggesting that there are many other factors besides this species’ larval potential to drift great distances that determine larval dispersal and consequent connectivity.

The most frequent *Ho. atra* haplotype, accounting for almost 58% of samples, is found in all four sampled locations, suggesting an overall trend of connectivity across the region despite observed differences in the degree of connectivity in each pair of sites. The boom-bust demographic cycle of *Ho. atra*^38^ may explain how all populations came to share the dominant haplotype, but still display levels of differentiation between populations. This shared haplotype may be the vestige of a time when there were large population sizes and high rates of migration, while the varying levels of genetic differentiation may be the result of subsequent population contraction and lesser gene flow across the archipelago. This is supported by *Ho. atra*’s positive and non-significant Tajima’s D, which indicates that the sea cucumber populations studied may have undergone recent demographic contraction^39^.

Results of the MIGRATE analyses were complex and idiosyncratic among all three species studied (Table 5). Gene flow patterns support a scenario of high connectivity for both fish species, with the magnitude of migrants exchanged among populations estimated at hundreds to thousands per generation. Patterns of directionality in *E. merra* and *Ha. trimaculatus* were complex and occasionally contradictory – for instance, Vanua Levu exported more migrants than it received from other locations in *E. merra*, whereas in *Ha. trimaculatus* the opposite was true. Both fish species also exhibited higher numbers of migrants coming from the western locality of Naviti and settling in the eastern island of Vanuabalavu than vice versa, indicating that western populations could be seeding eastern localities in spite of the prevailing westward-moving oceanic currents. *Ho. atra* shows a different pattern, in which the larvae seem to follow the oceanic currents resulting in the eastern island of Vanuabalavu exporting higher numbers of larval migrants to the western localities than vice versa. This concurs with previous studies showing unidirectional patterns of east-to-west gene flow in several taxa^28^. *Ho. atra*’s east to west pattern of gene flow is also supported by the haplotype diversities of each location, as the western population of Naviti, in the Yasawa islands, has the highest intra-population genetic diversity while Vanuabalavu, which lies farthest to the east in the Lau group, has the lowest. This suggests that larvae spawned in Nagigi, Taveuni, Vanuabalavu and other unsampled populations may potentially recruit more often to reefs in the western Yasawa Islands, promoting high genetic diversity. These contrasting patterns of gene flow observed between the fishes and the sea cucumber could be due to demographic stochasticity, differences in reproductive mode or timing, or seasonal variation in the flow of the Bligh Waters, which is common in other intra-pelagic currents^40^.

The dearth of literature on the inter- and intra-annual physical oceanography of the Fijian archipelago makes it difficult to assess the role of variation in currents on the data presented here. Genetic connectivity studies conducted in regions with better-studied oceanography have shown both inter-annual and intra-annual oceanographic features to be responsible for population connectivity or structure. Galarza *et al*.^41^ found oceanic fronts in the Mediterranean Sea to be associated with genetic structuring for several fish species, presumably due to the inability of larvae spawned on one side of the front to disperse across the front and admix with populations on the other side. Similarly, the Kuroshio Current in the East China Sea limits dispersal and therefore generates population differentiation between mudskippers in China and those in Japan^42^. Mesoscale eddies, which occur on a length scale of less than 100 km and last less than one month, have been implicated in keeping newly spawned larvae close to their natal site, as opposed to dispersing out into the open ocean^43^. Interpretations of genetic structuring and connectivity patterns found across species studied in the Fijian archipelago, as well as the idiosyncrasies in the data, almost certainly require a better understanding of the underlying oceanography. This clearly presents a priority for future research.

The Tajima’s D values for both *E. merra* and *Ha. trimaculatus* were strongly negative and while non-significant, they may indicate that both species may have undergone rapid population expansion in recent evolutionary history. Because the program MIGRATE assumes stable population dynamics, migration estimates can be strongly influenced by population expansion or contraction. Thus, the results of gene flow in the fish species should be taken with caution, particularly in the case of directionality. While the Tajima’s D value for *Ho. atra* was slightly positive, potentially indicating that this species has undergone a recent population bottleneck, the p-value for this estimate was much larger than those of the fishes and therefore likely does not have a significant effect on MIGRATE analyses^39^.

Another caveat of this study is its low sample sizes for *E. merra* at two locations (n = 10 and 12), and for *Ho. atra* at three locations (n = 10, 7, and 5). High amounts of nucleotide and haplotype diversity, like those found in *E. merra*, can sometimes be a relic of low sample sizes - however, high genetic diversity is a very common finding in marine fishes, and studies with very large sample sizes often observe high haplotype diversities as well^33^,^44^. Additionally, *Ha. trimaculatus* displayed almost identical measures of haplotype and nucleotide diversity, while having larger sample sizes than *E. merra* at nearly every location. Our power analyses show that with the sample sizes used for *E. merra* and *Ha. trimaculatus*, the diverse mtDNA control region confers low statistical power in detecting very weak levels of differentiation (**Φ**_ST_ of 0.01 or below), thus limiting our study’s ability to rule out a type II error within this range. However, the magnitude of differentiation below 0.01 is so low that even if our study did miss populations exhibiting partitioning at these levels, the conclusions drawn would be effectively the same – that both fish species exhibit high amounts of connectivity between their populations^31^.

In *Ho. atra*, on the other hand, this study observed very low haplotype diversities across all locations, even in Vanuabalavu where sample sizes were high (n = 18). While low sample sizes understandably lead to low observed genetic diversity, it is also likely that this species’ reproductive ecology (e.g. fission, or budding) has contributed to higher local densities of genetically identical individuals than in exclusively sexually reproducing species. Thus, while results for this species should certainly be taken with caution, it is possible that low sample sizes in this study may be capturing an adequate amount of the gene pool at each location.

Critiques for mtDNA-based phylogeographic studies often concern the variable rate of mutation observed between these markers and others – mtDNA often reach higher F_ST_ values than nuclear DNA^45^ – and the lack of power caused by high levels of diversity^46^. However, rapid mutation rates of mtDNA have been shown to provide high sensitivity for detecting genetic differentiation on short evolutionary time scales, rendering these markers highly useful for studies like this one investigating population-level connectivity^47^,^48^,^49^. In addition, a recent analysis has shown that mtDNA markers consistently display higher power to detect population divergence than any single nuclear marker when sample sizes are sufficient^50^. As a result, mtDNA sequences have been the markers of choice for marine connectivity studies in the Indo-Pacific for the past 20 years^51^. While multi-locus analyses certainly allow for a more nuanced evaluation of connectivity, it is not necessarily the case that adding nuclear loci would reveal significantly more information^52^.

Additionally, use of markers that have been used for previous phylogeography studies allows for easier comparison of results across taxa and locations^51^. The control region was used to analyse genetic connectivity in the only other study of fish biogeography in Fiji^28^, and thus we chose this ‘legacy marker’ for our analyses of the fish data. Similarly, the COI was used in a Hawai’ian biogeography study of *Ho. atra*^53^, so we chose to use the same marker in this study for the sake of comparison. Using relatively inexpensive markers and technologies for analyses in biogeography studies makes such studies more doable across underrepresented taxa and regions, and thus broadens the scope of known genetic connectivity patterns across the Pacific. This is especially critical from a management perspective.

## Conclusions and Management Implications

The high connectivity observed among populations of the fish species suggests that prioritizing one region over another for the placement of marine reserves in Fiji is not necessary for the conservation and persistence of *E. merra* and *Ha. trimaculatus* populations. Instead, each region sampled should be considered equally important for protection, as each node in these meta-populations contributes ecologically and economically significant amounts of migrants to the others. This finding provides managers with greater flexibility in conservation prioritization, as they can focus on areas that have strong local support for conservation, or areas like the Vatu-i-Ra seascape (between Viti Levu and Vanua Levu), which have been identified as having exceptional habitat quality^54^. Placing some portion of habitat within these regions under protection will promote the persistence and resilience of the entire system, while enabling fine-scale locations of protected areas to be determined by habitat representation, social and economic factors, or other aspects of reserve network design. This may help alleviate the tensions that often exist during marine spatial planning, between scientific information and social or economic priorities^55^.

Conversely, with *Ho. atra*, some populations are well connected to each other and others are isolated, which necessitates a more nuanced spatial management scenario. For instance, since the Vanua Levu and Taveuni populations are genetically differentiated, it is recommended that they be treated as separate management areas to preserve their genetic diversity, despite the fact that both lie within Fiji’s Northern Division. Additionally, since the Vanuabalavu population in the Lau archipelago to the east likely provides larval recruits and thereby genetic diversity to western reefs, this region should be a conservation priority for the management of the species. Upstream areas, such as Vanuabalavu for *Ho. atra* and Naviti for the fish, should be considered priorities for monitoring, as the health of downstream populations is largely reliant on the well-being of upstream areas.

Ultimately, our data suggest management at the larger Division or Provincial scale may be appropriate for species like *E. merra* and *Ha. trimaculatus* that exhibit high amounts of connectivity within the country, but for those like *Ho. atra* with low connectivity and high genetic partitioning among populations, management at the provincial or local scale may be required to ensure the persistence of isolated populations.

## Methods

### Study species

*Epinephelus merra* and *Halichoeres trimaculatus* are both widespread Indo-Pacific coral reef fishes, and are commonly targeted by fishermen in Fiji^56^,^57^. *E. merra* is typically caught by spear or by hook and line, and is most commonly used for subsistence and occasionally sold in fish markets. *Ha. trimaculatus* is caught primarily via net, and is more often used for subsistence or fed to livestock, depending on their size at harvest. Both species are found in protected seaward reefs and lagoonal areas, and both have small home ranges, rarely moving more than 1 km away from their adult home reefs^58^,^59^.

*Holothuria atra*, known as the black sea cucumber or the lollyfish, is a major commercial fisheries target throughout the Indo-Pacific^60^,^61^. *Ho. atra* can reproduce both sexually via broadcast spawning, and asexually by fissioning into anterior and posterior parts and then regenerating a full body from each part^38^,^62^,^63^. *Ho. atra*’s larval duration lasts approximately 18–25 days^53^,^64^, and its range extends from the Western Indian Ocean to the Eastern Pacific Ocean. Like the fishes, they tend to be relatively sedentary as adults with congeners traveling <20 m per day^65^.

### Ethics Statement

The study protocol was approved by the Columbia University Animal Care Committee (protocol no. AC-AAAF6300) and followed the laws of the Republic of Fiji and with permission of the traditional marine resource owners.

### Sample collection

**Figure 1:**
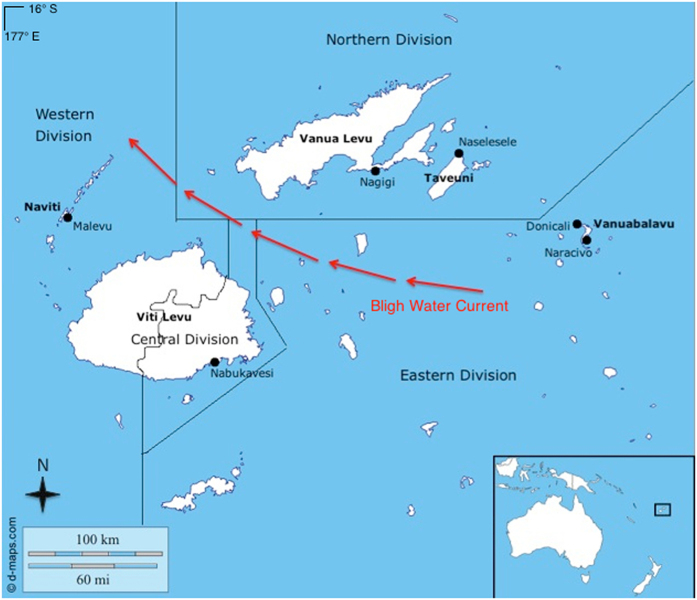
Sampling locations within Fiji, with island names in bold and village names adjacent to points. Division boundaries and path of the Bligh Water Current (denoted by red arrows) are approximate. Map © d-maps.com available at http://dmaps.com/carte.php?num_car=28136&lang=en

**Figure 2:**
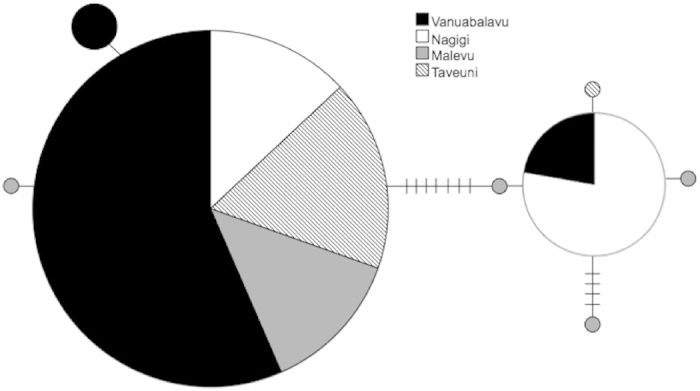
Haplotype network of *Ho. atra* samples. Each circle represents a haplotype. Size of the circle is scaled to the number of individuals that share that haplotype, Hash marks on connecting lines indicate the number of base pair differences between haplotypes. Shading represents the island where individuals were sampled.

**Figure 3:**
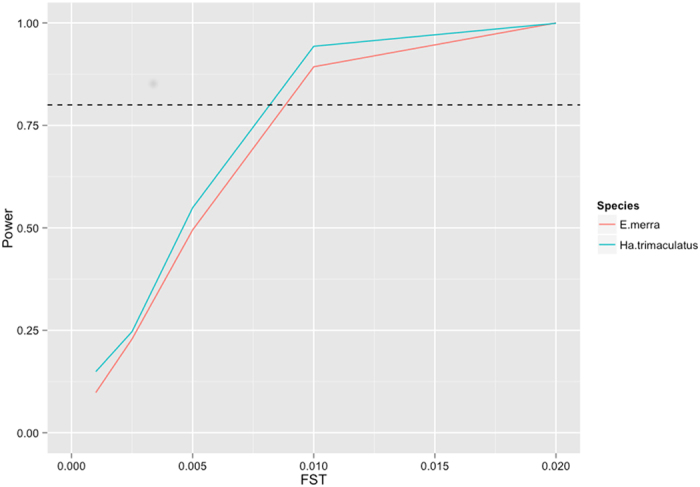
POWSIM analysis results showing power to detect structure among populations of *Epinephelus merra* and *Halichoeres trimaculatus* at different F_ST_ levels. Power is expressed as the proportion of significant outcomes after 1,000 replicates. Dashed line is at 0.80, the minimum acceptable power level put forth by^72^.

We sampled at six locations across Fiji (Fig. 1). Study sites were chosen to maximize regional coverage within the country, and sites were spread across the four Divisions of Fiji (Northern, Western, Central, and Eastern).

Fish were collected by net, spear or through purchase from local fishermen, while *Ho. atra* were collected by hand (Table 1). After samples were collected, gill clips from fish, or dermal clips from *Ho. atra* were taken and stored in ethanol or frozen in liquid nitrogen to preserve DNA. The whole samples were then fixed in formalin for accession into the ichthyology collections at the American Museum of Natural History.

### Genetic Analyses

Genomic DNA was extracted from gill clips and dermal samples using a DNeasy Tissue Kit (Qiagen, Hilden, Germany). For fish samples, we amplified the mitochondrial control region by PCR using the primers CR-A and CR-E^66^. For *Ho*. atra we amplified part of the cytochrome c oxidase 1 (COI) gene using primers designed by^53^. PCR parameters can be found in Supplementary Note S1 online. Sequences were generated on an ABI 3730 sequencer (Applied Biosystems, Inc., Foster City, CA, USA) and sequence quality was checked and sequences were aligned using Geneious 8.0^67^. Sequences were deposited in GENBANK with the following accession numbers: *E. merra* KT353114-KT353195, *Ha. trimaculatus* KT329098-KT329188, *Ho. atra* KT378456-KT378495.

### Data Analysis

To investigate the amount of genetic differentiation occurring between sampled populations, pairwise **Φ**_ST_ measures (a modified F-statistic specific to mitochondrial DNA) were calculated for each species in ARLEQUIN 3.5^68^, using 1000 replicates to estimate significance. Because of high haplotype diversities observed in the fish species and relatively low sample sizes, the statistical power of the control region marker was evaluated with POWSIM^69^, using a Ne of 5,000 and adjusting generation time (t) to assess power at multiple FST values (0.001, 0.0025, 0.005, 0.01, and 0.02). Power was expressed as the proportion of significant outcomes for 1,000 replicates.

To test whether regional-level genetic structuring was present, analyses of molecular variance (AMOVAs) were also performed in ARLEQUIN. For *E. merra*, each of the 6 sampling localities was initially treated as a separate entity, and when no significant differences were detected between the villages of Donicali and Naracivo (13 km apart on the island of Vanuabalavu), the data were pooled into island-level assemblages and reanalysed. Extremely low sample sizes for *Ha. trimaculatus* and *Ho. atra* in Donicali (n = 3 and n = 4, respectively) would have made individual locality results unreliable, so these two species were grouped at the island-level at the outset. Further, if no differentiation was detected between the island-level assemblages for each species, analyses were repeated with populations grouped by Division to investigate whether Fiji’s large-scale political boundaries are appropriate for the management of biological populations.

We calculated magnitude and direction of gene flow at the island-level using MIGRATE-n^70^. Two runs of 1,000,000 generations were conducted for each species with an initial 25% burn-in, Because of this program’s underlying assumptions that population size and migration rates have not changed over time, Tajima’s D statistics were determined in ARLEQUIN, to assess the likelihood of recent demographic expansion or contraction for each species.

To test whether any observed genetic differences could be due to geographic distance versus phylogeographic barriers, a Mantel test was also performed for each species using Isolation By Distance 3.23^71^.

## Additional Information

**How to cite this article**: Eastwood, E. K. *et al*. Population Connectivity Measures of Fishery-Targeted Coral Reef Species to Inform Marine Reserve Network Design in Fiji. *Sci. Rep*. **6**, 19318; doi: 10.1038/srep19318 (2016).

## Figures and Tables

**Table 1:** Geographic locations of sampling sites, and sample sizes for each species.

Division	Island (*Village*)	Latitude, Longitude	*Epinephelus merra*(N = 82)	*Halichoeres trimaculatus*(N = 91)	*Holothuria atra*(N = 40)
Northern	**Vanua Levu** (*Nagigi*)	S 16° 80’ 44.18” W 179° 48'05.53”	20	23	10
Northern	**Taveuni** (*Naselesele*)	S 16° 41’ 41.19” W 179° 52’ 1.52”	12	21	5
Western	**Naviti** (*Malevu*)	S 17° 6’ 54.12” E 177° 16’ 51.18”	11	16	7
Central	**Viti Levu** (*Nabukavesi*)	S 18° 13’ 20.42” E 178° 15’ 58.08”	16	15	–
Eastern	**Vanuabalavu** (*Naracivo*, *Donicali*)	S 17° 13’ 15.84” W 178° 57’ 53.57”	23	16	18

**Table 2:** Results of sequencing the mitochondrial control region, and larval durations for each study species^64^,^73^,^74^.

Species	Individuals	Haplotypes	π	Θs (SD)	Sequence Length	Larval Duration
*Epinephelus merra*	82	66	0.03	16.67 (4.51)	475	39 days
*Halichoeres trimaculatus*	91	75	0.03	13.97 (3.77)	376	27 days
*Holothuria atra*	40	8	0.01	3.18 (1.61)	376	18–25 days

**Table 3:** 

	Vanua Levu	Taveuni	Naviti	Vanuabalavu
**(A)** ***E. merra***Vanua Levu	–			
Taveuni	0.00000	–		
Naviti	0.05327	0.01336	–	
Vanuabalavu	0.00702	0.00000	0.00000	–
Viti Levu	0.00602	0.00000	0.00602	0.00000
**(B)** ***Ha. trimaculatus***Vanua Levu	–			
Taveuni	0.00547	–		
Naviti	0.00000	0.00000	–	
Vanuabalavu	0.00000	0.00000	0.00000	–
Viti Levu	**0.07232**	0.00196	0.02021	0.01350
**(C)** ***Ho. atra***Vanua Levu	–			
Taveuni	0.3287	–		
Naviti	0.0865	0.00000	–	
Vanuabalavu	**0.5042**	0.00000	0.1395	–

Pairwise Φ_ST_ values for. (**A**) E. merra (**B**) Ha. trimaculatus and (**C**) Ho. atra populations at the islandassemblage level in Fiji. Significant p-values are in bold.

**Table 4:** Analysis of molecular variance using the mitochondrial control region between

Source of variation	d.f.	Sum of squares	Percentage of variation	Statistics	*P*
**(A)** Among groups	4	22.069	−2.02	F_CT_ = −0.020	0.785
Among populations within groups	1	6.865	1.98	F_SC_ = 0.019	0.218
Within populations	76	437.262	100.04	F_ST_ = 0.000	0.424
**(B)** Among groups	3	17.781	1.59	F_CT_ = 0.016	0.298
Among populations within groups	1	4.288	−1.71	F_SC_ = −0.017	0.617
Within populations	77	444.127	100.13	F_ST_ = −0.001	0.490
**(C)** Among groups	3	14.387	−0.10	F_CT_ = −0.001	0.723
Among populations within groups	1	5.034	0.61	F_SC_ = 0.006	0.295
Within populations	85	377.046	99.49	F_ST_ = 0.005	0.281

**(A)** five groups of *E. merra* (Naviti, Vanua Levu, Taveuni, Vanuabalavu, Viti Levu) reflecting island-level assemblages, **(B)** four groups of *E. merra* (Northern Division, Eastern Division, Western Division, Central Division) reflecting political boundaries, and **(C)** four groups of *Ha. trimaculatus* (Northern Division, Eastern Division, Western Division, Central Division) reflecting political boundaries. Negative values are presented, but are effectively equal to zero.

**Table 5:** Results of MIGRATE analysis for each species.

Species		Vanua Levu	Taveuni	Naviti	Vanuabalavu	Viti Levu
*E. merra*	Vanua Levu	–	5850.0	543.3	896.7	576.7
	Taveuni	3183.3	–	276.7	1516.7	1256.7
	Naviti	7490.0	2676.7	–	656.7	690.0
	Vanuabalavu	6536.7	2436.7	190.0	–	2023.3
	Viti Levu	5530.0	1403.3	116.7	1790.0	–
*Ha. trimaculatus*	Vanua Levu	–	296.7	916.7	1683.3	1050.0
	Taveuni	523.3	–	683.3	450.0	1330.0
	Naviti	736.7	450.0	–	1636.7	3930.0
	Vanuabalavu	316.7	430.0	623.3	–	1936.7
	Viti Levu	550.0	516.7	1123.3	1250.0	–
*Ho. atra*	Vanua Levu	–	522.3	479.7	555	–
	Taveuni	455	–	514.3	411.7	–
	Naviti	438.3	487	–	388.3	–
	Vanuabalavu	533	672.3	582.3	–	–

Median number of recruits per generation is shown, with migration occurring from row to column.
